# A Literature Review of Simulation-Based Nursing Education in Korea

**DOI:** 10.3390/nursrep13010046

**Published:** 2023-03-19

**Authors:** Sumee Oh, Jungmin Park

**Affiliations:** College of Nursing, Hanyang University, Seoul 04763, Republic of Korea; ohsumee@hanyang.ac.kr

**Keywords:** literature review, nursing simulation, nursing student, simulation-based education, simulation-based education in the Republic of Korea

## Abstract

This study reviewed the papers that studied the effect of simulation nursing education in the nursing field and examined the trend of simulation nursing education for nursing college students in Korea. Background: Simulation-based education started receiving attention as a pedagogical method in order to provide medical service of high quality in an ethical and safe environment. This has been of great importance during the coronavirus disease 2019 global pandemic. This literature review was conducted to suggest a direction for simulation-based nursing education in Korea. Methods: For literature searches, the authors used the following search terms in the Web of Science, CINAHL, Scopus, PubMed—‘utilization’, ‘simulation,’ ‘nursing student’, ‘nursing education’. A final search was conducted on 6 January 2021. The materials for this study were collected through literature searches according to the PRISMA guidelines. Results: 25 papers were selected as the final literature for analysis. The study was conducted for 48 percent of senior students in nursing college students in Korea (N = 12). High fidelity (HF) as the simulation type was 44 percent (N = 11). The simulation education subjects were composed of 52 percent adult health nursing (N = 13). According to educational goals described by Benzamine Bloom (1956), 90% in the psychomotor domain is considered a positive learning achievement. Conclusions: Effectiveness in the psychomotor domain through simulation-based training is correlated with expert nursing. It is essential to develop a systematic debriefing model and methods to evaluate performance and learning in the short- and long-term to expand the effectiveness of simulation-based education in nursing.

## 1. Introduction

Both the increasing severity of a patient’s condition and the complicated and changing medical environment require high-quality medical services to ensure the best patient outcomes [1]. There is a growing need for good-quality educational courses in nursing colleges to educate nursing experts who are equipped with proper certifications. Such nurses who receive an advanced nursing education can help reduce the delays in recovery and damaging side effects for patients, thereby preventing failure during treatment. By reducing mortality rates, nurses will be able to play a vital role in treatment and even patient satisfaction by helping patients and their families avoid unnecessary medical costs. It is difficult even for well-educated nurses to be fully prepared for nursing [2]. With the recent increase in the awareness of patients’ rights and professional ethics of healthcare professionals, there are limited opportunities for gaining practical experience in real-world clinical settings [3]. Therefore, changes are needed in nursing education to enable students to acquire the necessary knowledge and skills [4]. Owing to the limitations of clinical practice, simulation-based education has become a promising alternative, enabling nursing professionals and students to gain and develop skills without facing patients directly. Efforts have been made to ensure that simulation-based education provides good-quality and effective education [5]. The outbreak of coronavirus disease 2019 (COVID-19) caused enormous damage to learning opportunities for students in the medical environment. Isolation, social distancing, and society’s need for prepared clinical nurses created major challenges for nursing education [6]. The appearance of new infective diseases, such as COVID-19 and Middle East respiratory syndrome, has created the requirement for new pedagogical methods for nurses instead of the traditional methods in practice [7,8]. Simulation-based education applies a patient care scenario in a safe educational environment, which will likely reappear in clinical settings, to help nurses gain skills and experience managing the situation, thereby reducing error in real-world clinical situations [9]. Nursing students enjoy the simulations based on feedback to increase their self-confidence and satisfaction [10]. In domestic nursing education, learning methods that use standardized patients (SPs) as a teaching method have been adopted since 2001 [11]. Simulation-based education started in 2006 and has increased rapidly since then [12]. The Korea Accreditation Board of Nursing Education established a requirement for 1000 h of simulation-based practical experience per year for training expert nurses as part of the nursing education accreditation evaluation [13]. Literature reviews were conducted to determine how simulation-based education has been used in Korea for nurses and nursing students from 2014 to 2020 [12,14]. With the increase in simulation-based practical education in the curriculum of nursing colleges, it is essential to review the effectiveness and the direction of simulation-based education in nursing. Hence, this study will suggest directions for simulation-based nursing education in Korea after understanding trends and the effect of this type of education on the field.

## 2. Methods

### 2.1. Aim

This study examines reviews conducted on simulation-based education as a subject in the national curriculum for nursing students in Korea for trends and outcomes.

### 2.2. Design

A literature review was conducted to suggest directions for simulation-based nursing education in Korea.

### 2.3. Search Methods

The materials for this work were collected according to the PRISMA guidelines [15].

**Figure 1:**
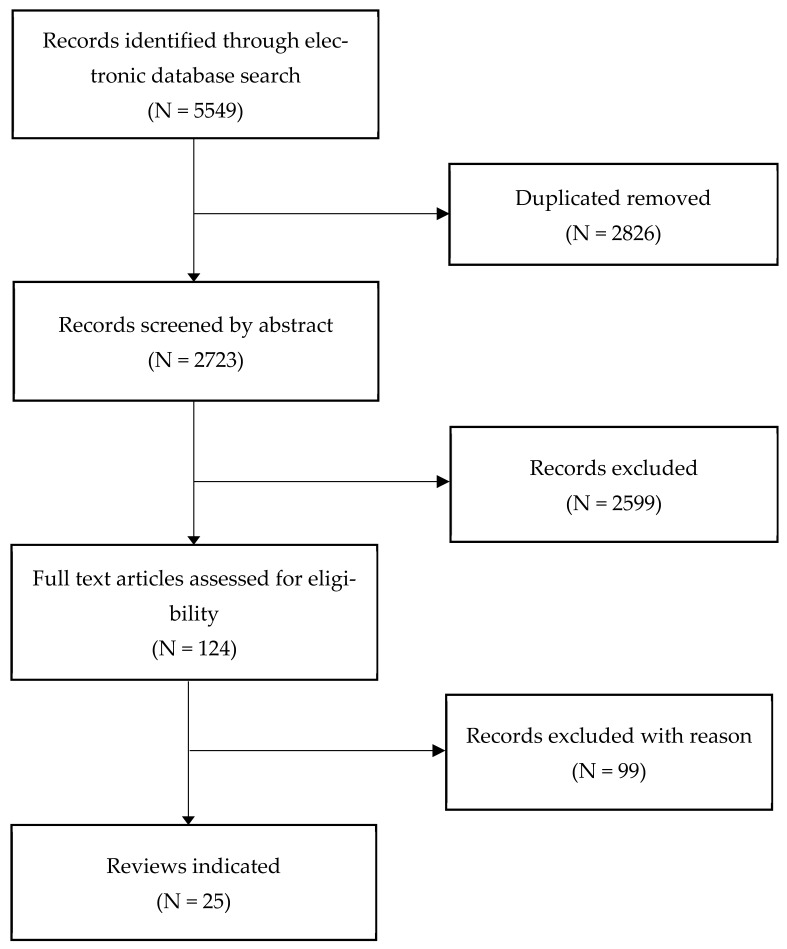
Search flow.

This work reviewed the effect of simulation nursing education on the nursing field and identified the trend of simulation nursing education for nursing college students in Korea. For the literature searches, the following search terms were entered in the Web of Science, CINAHL, Scopus, PubMed—‘utilization’, ‘simulation’, ‘nursing student’, ‘nursing education’. A final search was conducted on 6 January 2021. Based on the standard for certification from the Korean Accreditation Board of Nursing Education in 2014, practical simulation experience accounts for 10% of the total number of clinical practice hours for the graduation. These reviewed papers were limited to those published between 2014 and January 2021 and included a total of 5549 papers. Of these, 124 were excluded because they were dissertations, and 2723 were excluded because they were duplicated papers. Other bases for exclusion were papers that excluded clinic nurses or included overseas nursing college students, research conducted with the other students, simulation theory development studies, simulation education program evaluation, and simulation conception analyses. Finally, 25 papers were selected as the final literature for analysis (Figure 1).

### 2.4. Material Analysis Method

General features related to simulation, measured variables, and results in the selected twenty-five papers as study objects were analysed. The publication year of the study, the study subject, and study design type were analysed as general features. Other features were the simulation type and subject, as well as measured variables and the effects of the research. The selected papers were noted for acquiring high study quality and were reviewed in academic journals. The study collection and data extraction process was reviewed and finally agreed upon by two researchers through several meetings; there were no disagreements.

## 3. Results

### 3.1. Literature Selection

The goals and design, measured variables, mediation, and results of the selected simulation studies for Korean nursing college students appear in Table 1.

### 3.2. Study Design of Simulation-Based Education Studies

General features of simulation-based studies conducted since 2014 on Korean nursing students as subjects appear in Table 2.

Simulation-based nursing education studies in Korea were confirmed to comprise seven studies (28%) from 2014 to 2015, eight studies (32%) from 2016 to 2017, six studies (24%) from 2018 to 2019, and four studies (16%) in 2020. There have been no studies conducted on the curriculum for first year students of nursing colleges. The curriculum for simulation-based nursing education for students ranging from sophomores to junior year students was confirmed to include 13 studies (52%) and 12 studies (48%) for senior year students. Random-contrast experimental studies were part of 6 papers (24%), while those featuring a single group comprised 8 studies (32%); 11 studies featured similar control groups (44%). 

The analysis results for simulation relational variables appear in Table 3.

The simulation type was high fidelity (HF) in 11 cases (44%), standardized patient in 2 cases (8%), role-play (RP) in 5 cases (20%), low fidelity (LF) in 3 cases (12%), HF and SP in 1 case (4%), LF and SP in 1 case (4%), virtual reality (VR) and HF in 1 case (4%), and a Web-based study of HF in 1 case (4%). 

The analysis results categorized by the simulation subject courses of the papers appear in Table 4.

Adult health nursing was analysed in 13 studies (52%), pediatric nursing was examined in 6 studies (24%), maternity nursing in 2 studies (8%), mental health nursing in 1 study (4%), emergency nursing in 2 studies (8%), and other research in 1 section (4%).

### 3.3. A Related Measurement Variable and Results of Simulation-Based Education Research

According to educational goals described by Benzamine–Bloom (1956), the education goal classification areas were divided into cognitive, affective, and psychomotor domains (Table 5) [41,42]. The measurement variables of the cognitive domain were apprehension (one case), clinical judgment (three cases), communication ability (two cases), communication clarity (three cases), critical thinking skills (seven cases), knowledge (nine cases), learning effect (one case), metacognition (two cases), and problem-solving ability (three cases). The measurement variables of the affective domain were attitude (8 cases), collective (team) efficacy (2 cases), interest (1 case), perception (2 cases), satisfaction (10 cases), self-assertiveness (1 case), self-confidence (6 cases), self-efficacy (3 cases), self-leadership (1 case), difficulty (1 case), and stress (3 cases). The psychomotor domain consisted of skill performance (three cases), critical performance (three cases), confidence in performance (one case), clinical competency skill (one case), and practice (one case).

## 4. Discussion

Simulation provides a safe learning environment for the learner to develop the skills and behaviours necessary for effective nursing practice [43]. In addition, it improves clinical skills, reinforces learning through practiced actions ,and increases self-confidence [44]. Thus, after 2014 when a certification evaluation standard of the Korea Accreditation Board of nursing education had been implemented, research on the effectiveness of simulation-based education has been continuously progressing. However, the significance of simulation-based education has eluded national nursing education. An examination of the simulation-based curriculum revealed that while simulation-based subjects were included in the curriculum for sophomores and juniors, they carried more weight for seniors. One of the goals of simulation-based education is to equip nurses with the capacity to handle various situations in clinical settings [45,46]. Additionally, simulation-based education has been a crucial alternative to supplement clinical field practice during the pandemic. A study of a similar experiment by a contrast group revealed the most important weight in eleven elements (44%). Sample size justification was checked for all except four elements in 21 research projects. Except for one element, 24 elements appeared in instrument validation, demonstrating that verified invalidity changed the results. However, as the method used by Kim et al. is not valid, the results must be interpreted carefully. In a study containing 11 elements (44%), HF was the most common type of simulation. High-fidelity simulation (HFS) simulates potentially far deeper cognition by having the learner participate in the learning. In addition, HFS, by providing the experience of high concentration, has been recognized as an effective learning method for medicine. Another study mixed more than two types of simulations. Combining more than two types of methods helps understand complicated clinical fields. For effective simulation-based education, of student learning was assessed through structured self-examination and feedback [47]. Due to the increasing importance of debriefing, a simulation course was implemented by including debriefing in 19 research studies (76%). However, we lack a standardized debriefing model; one must be developed and applied for more effective simulation-based education. One study to compare with this study notes that simulation-based education has been expanding in other areas, such as pediatric, maternity, and emergency nursing [48]. However, this study can verify that adult health nursing comprises 13 studies (52%), accounting for the most weight [49,50].

The frequency of measuring knowledge was the highest, with a positive result of 70% in the evaluation area, which is related to the cognitive domain [42]. In the affective domain, the frequency of evaluating satisfaction was the highest and showed a positive result of 70% [42].

Simulation training in most nursing courses covers pediatric, adult, mental health, and maternal nursing [51]. Simulation-based education satisfies the criteria of a cognitive domain similar to a traditional learning method and meets the learning needs of students in the affective domain. Nonetheless, the evaluation method of the cognitive and affective domains is the most important assessment, which can take place through a survey using self-reporting forms. To achieve effective learning goals, it is important to implement various methods of evaluation. A positive learning achievement of 90% in the psychomotor domain implied that the lessons learned from simulation could be re-enacted in the clinical field. Additionally, through repeated education with increasing skill levels, the psychomotor domain showed the best correspondence to the goals of simulation-based education as a learning strategy for increasing clinical competency [52,53,54].

An area evaluation of education goal classification by Bloom showed a similar tendency in an HF precedence study, which likewise showed the outcome of qualified increase with higher levels of thinking [42]. Simulation-based education is a positive learning method for clinical judgment training and critical thinking, offering a safe environment for improving nursing knowledge and competency. Moreover, it is effective in removing the anxiety of the learner and encouraging teamwork [9,55,56]. Therefore, it is essential to develop simulation-based education that uses various themes. It is also crucial to develop methods to estimate the effectiveness of simulation-based education in the short- and long-term. Simulation-based nursing education should expand from the existing nursing college curriculum and work to reach more nursing students and professionals. Finally, a change in instruction methods can play an important role in nursing education.

## 5. Conclusions

Due to an increase in the severity of disease, the significance of simulation-based nursing education is expanding gradually with the increasing need for skilled expert nurses. Simulation-based education has been continuously progressing in recent years. However, simulation-based nursing education has been restricted to senior students. The simulations have concentrated on adult health nursing. In addition, there is either no mention of debriefing in simulation-based nursing education, or the debriefing is deficient. Further, effectiveness in the psychomotor domain was identified as the highest expert nursing skill that can be developed through simulation-based education. To improve the educational effectiveness in the psychomotor domain through simulation learning, we suggest that the learning participants should be expanded from seniors to juniors. Moreover, repeated simulation-based education performance and research on the effectiveness of simulations are important in long-term learning, for example, through the development of a systemic debriefing model.

## Figures and Tables

**Table 1:** Goal and design, measured variables, mediation, and outcomes.

Authors (Year)	Study Design	Sample Size (*n*), Sample Size Justification,	Instrument Validation, Debriefing	Outcome Variables
Roh and Lim, 2014 [16]	QE (without CG)	[Sample size (*n*)] 284 [Sample size justification] +	[Instrument Validation] + [Debriefing] +	[Pre-Course Simulation with an Emergency Nursing Clinical Course] Satisfaction (+)
Roh et al., 2014 [17]	QE (without CG)	[Sample size (*n*)] 185 [Sample size justification] −	[Instrument Validation] + [Debriefing] +	[Integrated Problem-Based Learning and Simulation Course] College-based stress (−) Student perceptions (−)
Shin and Kim, 2014 [18]	QE (with CG)	[Sample size (*n*)] 95 [Sample size justification] +	[Instrument Validation] + [Debriefing] +	[Pediatric Nursing Practicum Integrated Simulation Courseware] Critical (+) Clinical judgment (+) Simulation satisfaction (+)
Ahn and Kim, 2015 [19]	RCT	[Sample size (*n*)] 69 [Sample size justification] +	[Instrument Validation] + [Debriefing] +	[Simulation Activity and Debriefing Group] Self-confidence (±) Critical thinking skills (±)
Kang et al., 2015 [20]	QE (With CG)	[Sample size (*n*)] 205 [Sample size justification] +	[Instrument Validation] + [Debriefing] +	[Simulation Combined with PBL] Knowledge (+) Confidence in skill performance (−) Satisfaction (−)
Kim, H.Y and Kim, H.R., 2015 [21]	QE (With CG)	[Sample size (*n*)] 149 [Sample size justification] +	[Instrument Validation] + [Debriefing] +	[Colonoscopy-Based Simulation Education Program] Knowledge (+) Critical performance (+)
Shin et al., 2015 [22]	QE (With CG)	[Sample size (*n*)] 237 [Sample size justification] −	[Instrument Validation] + [Debriefing]	[Simulation Courseware on Critical Thinking in Undergraduate Nursing Students] Critical thinking (+)
Kang et al., 2016 [23]	RCT	[Sample size (*n*)] 74 [Sample size justification] +	[Instrument Validation] + [Debriefing] +	[Simulation with TBL] Lower achiever: Learning attitude (−), outcome (+) Higher achiever: Learning attitude (−), outcome (−)
Kim and Shin, 2016 [24]	RCT	[Sample size (*n*)] 47 [Sample size justification] +	[Instrument Validation] + [Debriefing] +	[Sex Education S-PBL Program] Gender role perception (−) Knowledge (+) Sexual attitude (+)
Chu and Hwang, 2017 [25]	QE (With CG)	[Sample size (*n*)] 144 [Sample size justification] +	[Instrument Validation] [Debriefing] +	[High-Fidelity Group] Self-efficacy (+) Problem-solving ability (+) Interest in learning (±) Level of stress (−) Satisfaction (+) Level of difficulty (−)
Jeong et al., 2017 [26]	QE (without CG)	[Sample size (*n*)] 70 [Sample size justification] +	[Instrument Validation] + [Debriefing]	[Senior Simulation Program] Attitudes toward seniors (+)
Jeong and Choi, 2017 [27]	QE (With CG)	[Sample size (*n*)] 48 [Sample size justification] +	[Instrument Validation] + [Debriefing] +	[Simulation, Debriefing Based on Clinical Judgment Model] Knowledge (+) Skill performance (+) Clinical judgment (+) Self-confidence (+) Satisfaction (−)
Lee et al., 2017 [28]	QE (With CG)	[Sample size (*n*)] 127 [Sample size justification] +	[Instrument Validation] + [Debriefing]	[Pre-education Combined with a Simulation for Caring for Children with Croup] Knowledge (+) Confidence in performance (+) Ability in nursing practice (+) Satisfaction (+)
Lee et al., 2017 [29]	QE (without CG)	[Sample size (*n*)] 176 [Sample size justification] −	[Instrument Validation] + [Debriefing]	[S-PBL Program] Metacognition (+) Team efficacy (−) Learning attitude (−)
Yu and Kang, 2017 [30]	QE (With CG)	[Sample size (*n*)] 62 [Sample size justification] +	[Instrument Validation] + [Debriefing]	[Role-Play Simulation Program Involving the SBAR Technique] Communication: score (+), clarity (+) Confidence (+) Satisfaction (−)
Kim and Park, 2018 [31]	QE (without CG)	[Sample size (*n*)] 86 [Sample size justification] +	[Instrument Validation] + [Debriefing]	[Simulation Practice] Stress (−) Self-esteem (−) Collective efficacy (+)
Kim and Yun, 2018 [32]	RCT	[Sample size (*n*)] 67 [Sample size justification] +	[Instrument Validation] + [Debriefing] +	[System Thinking-Based Simulation Program for Congestive Heart Failure] Critical thinking (−) Problem-solving ability (+) Clinical competency skills (+)
Kim et al., 2018 [33]	QE (without CG)	[Sample size (*n*)] 82 [Sample size justification] +	[Instrument Validation] + [Debriefing]	[Simulation Education with PBL] Communication apprehension (−) Self-assertiveness (−) Nursing clinical self-efficacy (+) Satisfaction (+)
Chae, 2019 [34]	QE (With CG)	[Sample size (*n*)] 60 [Sample size justification] −	[Instrument Validation] − [Debriefing] +	[SBAR Lectures Group] Communication clarity (+) Self-leadership (+) Attitude toward patient safety (+) Safety care performance (+)
Kim et al., 2019 [35]	QE (With CG)	[Sample size (*n*)] 48 [Sample size justification] +	[Instrument Validation] + [Debriefing] +	[ Blended Simulation of Care for Paediatric Patients with Asthma] Critical thinking (+) Problem-solving process (+) Critical performance (+)
Yun and Choi, 2019 [36]	RCT	[Sample size (*n*)] 104 [Sample size justification] +	[Instrument Validation] + [Debriefing] +	[Integration Sequences of S-PBL] Knowledge (+) Clinical performance (+) Clinical judgement (+) Self-confidence (−) Satisfaction (−)
Hwang and Kim, 2020 [37]	QE (without CG)	[Sample size (*n*)] 87 [Sample size justification] +	[Instrument Validation] + [Debriefing] +	[Home-Visit Simulation Scenario] Self-efficacy (+) Critical thinking (+) Learning effect (+) Self-confidence (+)
Jeong and Kim, 2020 [38]	RCT	[Sample size (*n*)] 54 [Sample size justification] +	[Instrument Validation] + [Debriefing] +	[SBAR Simulation Program] Knowledge (−) Skills (−) Attitude (−) Communication ability (−) Communication clarity (+)
Kim and Lee, 2020 [39]	QE (without CG)	[Sample size (*n*)] 34 [Sample size justification] +	[Instrument Validation] + [Debriefing] +	[Simulation-Based Education Program for Nursing Students Responding to Mass Casualty Incidents] Knowledge (−) Attitude (+) Satisfaction (+)
Son, 2020 [40]	QE (With CG)	[Sample size (*n*)] 78 [Sample size justification] +	[Instrument Validation] + [Debriefing] +	[S-PBL in Maternity Nursing Clinical Practicum] Learning attitude (+) Metacognition (−) Critical thinking (+)

Note. In the study, if the sample size, sample size justification, and instrument validation were used, I marked them with a plus sign. Although the three items were in the study, if the three items were not used in the study, I marked them as a minus. However, if the study did not mention the three items at all, I marked it as a blank. RCT: randomized controlled trial; QE: quasi-experimental; CG: control group; PBL: problem-based learning; SBAR: situation, background, assessment, and recommendation; S-PBL: simulation, problem-based learning; TBL: team-based learning; (±): not measuring; (+): relevance confirmed; (−): not relevant.

**Table 2:** General features of simulation studies (*n* = 25).

Categories	Variables	*n* (%)
Year	2014~2015	7 (28)
	2016~2017	8 (32)
	2018~2019	6 (24)
	2020	4 (16)
Participants	Freshmen	0 (0)
	Sophomore or Juniors	13 (52)
	Seniors	12 (48)
Study design	Randomized Controlled Trial	6 (24)
	Quasi-Experimental (without control group)	8 (32)
	Quasi-Experimental (with control group)	11 (44)

**Table 3:** Analysis results related to simulation relational variables of studies (*n* = 25).

Variables	Categories	*n* (%)
Type of simulation	HF	11 (44)
	LF	3 (12)
	RP	5 (20)
	SP	2 (8)
	SP and HF	1 (4)
	SP and LF	1 (4)
	VR simulation and HF	1 (4)
	Web-based HF	1 (4)

Note: HF: high fidelity; LF: low fidelity; RP: role-play; SP: standardized patient; VR: virtual reality.

**Table 4:** Analysis results classified by the simulation subject courses (*n* = 25).

Variables	Categories	*n* (%)
Type of subjects	Adult health nursing	13 (52)
	Pediatric nursing	6 (24)
	Maternity nursing	2 (8)
	Mental health nursing	1 (4)
	Emergency nursing	2 (8)
	Others	1 (4)

**Table 5:** Measurement variables and results of simulation-based education research (*n* = 25).

Categories	Variables	Number of the Variables	Number of the Variables Improvement of Outcome
Cognitive domain	Apprehension	1	0
	Clinical judgment	3	3
	Communication ability	2	1
	Communication clarity	3	3
	Critical thinking skill	7	6
	Knowledge	9	6
	Learning effect	1	1
	Metacognition	2	1
	Problem-solving ability	3	3
**Total**		31	24
Affective domain	Attitude	8	5
	Collective (team) efficacy	2	1
	Interesting	1	1
	Perception	2	0
	Satisfaction	10	6
	Self-assertiveness	1	0
	Self-confidence	6	4
	Self-efficacy	3	3
	Self-leadership	1	1
	Difficulty ^a^	1	0 (1)
	Stress ^a^	3	0 (3)
**Total**		38	25
Psychomotor domain	Skill performance	3	2
	Critical performance	3	3
	Confidence in performance	1	1
	Clinical competency skill	1	1
	Practice	1	1
**Total**		9	8

Note: ^a^ specific notes appear in reverse.
